# Genetic Engineering of *Dictyostelium discoideum* Cells Based on Selection and Growth on Bacteria

**DOI:** 10.3791/58981

**Published:** 2019-01-25

**Authors:** Peggy Paschke, David A. Knecht, Thomas D. Williams, Peter A. Thomason, Robert H. Insall, Jonathan R. Chubb, Robert R. Kay, Douwe M. Veltman

**Affiliations:** ^1^MRC Laboratory of Molecular Biology; ^2^Department of Molecular and Cell Biology, University of Connecticut; ^3^Cancer Research UK Beatson Institute Glasgow; ^4^MRC Laboratory for Molecular Cell Biology, University College London; ^5^Department of Cell and Developmental Biology, University College London

**Keywords:** Genetics, Issue 143, *Dictyostelium*, transfection, knock-out, knock-in, extrachromosomal plasmids, *act5*, overexpression, chemotaxis, motility, macropinocytosis

## Abstract

*Dictyostelium discoideum* is an intriguing model organism for the study of cell differentiation processes during development, cell signaling, and other important cellular biology questions. The technologies available to genetically manipulate *Dictyostelium* cells are well-developed. Transfections can be performed using different selectable markers and marker re-cycling, including homologous recombination and insertional mutagenesis. This is supported by a well-annotated genome. However, these approaches are optimized for axenic cell lines growing in liquid cultures and are difficult to apply to non-axenic wild-type cells, which feed only on bacteria. The mutations that are present in axenic strains disturb Ras signaling, causing excessive macropinocytosis required for feeding, and impair cell migration, which confounds the interpretation of signal transduction and chemotaxis experiments in those strains. Earlier attempts to genetically manipulate non-axenic cells have lacked efficiency and required complex experimental procedures. We have developed a simple transfection protocol that, for the first time, overcomes these limitations. Those series of large improvements to *Dictyostelium* molecular genetics allow wild-type cells to be manipulated as easily as standard laboratory strains. In addition to the advantages for studying uncorrupted signaling and motility processes, mutants that disrupt macropinocytosis-based growth can now be readily isolated. Furthermore, the entire transfection workflow is greatly accelerated, with recombinant cells that can be generated in days rather than weeks. Another advantage is that molecular genetics can further be performed with freshly isolated wild-type* Dictyostelium* samples from the environment. This can help to extend the scope of approaches used in these research areas.

**Figure:**
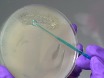


## Introduction

The *Dictyostelium* genus are soil-living social amoeba that mainly feed on bacteria. Being placed in the phylum Amoebozoa, a large number of species have been isolated that can be grouped into four different clades^1^. The species *Dictyostelium discoideum* (*D. discoideum*) has become a popular model organism to study complex cellular processes such as cell migration and phagocytosis. To control and standardize experimental conditions, axenic cell lines have been developed that are able to grow in complex or defined liquid medium in the absence of bacteria^2^. Of particular importance are the Ax2, Ax3, and Ax4 strains, which were all generated in the 1970s and ultimately derived from a single wild isolate NC4^3^. Tools for genetic engineering were developed in these axenic strains, resulting in the first published knockout in 1987^4^,^5^. Protocols were further developed and optimized for use under axenic conditions^6^,^7^.

Adaptation of these protocols to wild-type *D. discoideum* strains that are not able to grow in liquid broth have been attempted by several laboratories. However, this has not become fully successful since the transfection protocols are complex and lack efficiency, in part due to the capacity of the bacteria to act as a sink for the selective reagents^8^,^9^. As a result, essentially all molecular data on *D. discoideum* comes from descendants of a single wild-type isolate. We wanted to overcome this limitation and develop a method to genetically modify *D. discoideum* cells independent of their ability to grow in liquid medium. The need for such a method can be explained by the observation that it was assumed in the past that the mutations allowing axenic growth were mainly neutral and did not impair cell physiology. This supposition is only partially correct. In general, there are two notable differences; first, between the different isolated axenic strains, and second, when these axenic strains are compared with non-axenic wild isolates^8^,^9^.

Perhaps the most critical factor is the key axenic gene, *axe*B, that was identified recently as RasGAP NF1. The major function of NF1 as a RasGAP is to restrain Ras activity^3^. The deletion of the enzyme in all axenic strains leads to excessive Ras activity manifested as the formation of large active Ras patches. These enlarged Ras patches lead to the accumulation of PIP3 in the plasma membrane. Those coincidental appearing patches of PIP3 and active Ras are a template for the formation of a circular ruffle that eventually closes and leads to the formation of macropinosomes^10^. The consequence is an excessive increase in macropinocytic activity. Macropinocytosis is an actin-driven process. A competition for cytoskeletal components for the formation of either macropinosomes or pseudopods is the result. Its impact on cell behaviour is reflected in the almost complete prevention of chemotaxis of vegetative cells to folate^11^. The massively enlarged PIP3 patches are very persistent. Even in starved cells, the PIP3 patches remain and can be misinterpreted as pseudopods, which can cause problems interpreting studies on chemotaxis to cAMP.

In some cases, the NF1 mutation is experimentally useful. This leads us to a second motivation for developing a transfection method for bacterially grown *D. discoideum* cells, since the increase in macropinocytosis rate makes axenic cells valuable for investigating fundamental aspects of this process^12^. However, mutation in the genes required for macropinocytosis, such as Ras and PI3-kinases^10^, have almost abolished axenic growth, making it necessary to manipulate these cells through growth on bacteria. Another reason that renders bacteria-based transfections valuable is the increasing use of Dictyostelids to explore questions in the evolution of multi-cellularity^13^,^14^, kin recognition^15^,^16^, and altruistic cellular behaviour, which mainly depend on the use of freshly isolated wild-type isolates^17^. All mentioned research areas can be facilitated by efficient methods for genetic manipulation of wild isolates, which are non-axenic and do not grow in liquid broth.

Our protocols allow for overcoming of the described limitations. Taken together, the possibility of performing genetic manipulations with bacterial grown *D. discoideum* cells holds benefits for all *Dictyostelium* researchers, even if it is just the increased speed of the selection process due to faster growth of the amoeba (4 h doubling time) on bacteria compared to growth in axenic media (10 h doubling time).

## Protocol

### 1. Preparation of Cells and Materials

1. **SorMC buffer preparation**
  1. Prepare 100 mL of 100x SorMC (Sorensen buffer including MgCl_2_ and CaCl_2_) buffer by dissolving 20.36 g of KH_2_PO_4 _(15 mM) and 5.47 g of Na_2_HPO_4_·7 H_2_O (2mM) in 100 mL of ddH_2_O water. Stir the solution at room temperature (RT) and bring the volume to 100 mL with dH_2_O. **NOTE:** The resulting buffer has a pH of 6 and does not need further adjusting.
  2. Produce 1000 mL of 1x working solution in ddH_2_O. Add 50 µL each of MgCl_2 _and CaCl_2 _to achieve final concentrations of 50 µM each. Filter sterilize the solution using a 0.22 µm filter. **NOTE:** Always add MgCl_2 _and CaCl_2_ to the 1x buffer to avoid precipitation of the salts in the 100x stock solution.
  3. Alternatively, prepare KK_2_ buffer (2.2 g of KH_2_PO_4_ and 0.7 g of K_2_HPO_4_ for 1 L of buffer) supplemented with 50 µM MgCl_2_ and 50 µM CaCl_2_ (herein referred to as KK_2_MC). Use this buffer throughout instead of SorMC.
2. **Preparation of bacteria as food source for *D. discoideum***
  1. Use a single colony of *K. aerogenes* and inoculate 1 L of LB-medium (lysogeny broth). Use a 2 L flask. Let bacteria grow overnight at 37 °C with shaking at 220 rpm. **NOTE:** If large amounts of bacteria are required, use richer media like 2xTY (yeast extract tryptone medium) or SOB (super optimal broth) instead of LB. In case the use of *K. aerogenes* bacteria is not permitted due to safety restrictions, BL21 *E. coli* can be used instead.
  2. Harvest the cells the next day by spinning them down in two 500 mL centrifuge tubes at ~6,600 x *g *for 20 min. Wash bacteria once with 500 mL of SorMC buffer.
  3. Resuspend the pellet in 20 mL of SorMC. Check the OD_600_ (optical density at 600 nm) using a photometer. Dilute with the same buffer to an OD_600_ of around 100. **NOTE:** *K. aerogenes* bacteria are difficult to pellet, so a relatively high speed for spinning down the bacteria is necessary to avoid loss of food bacteria. The resulting bacterial stock solution can be stored for up to 4 months in the fridge at 4 °C and maintain its utility as food source for *D. discoideum*. 1 L of overnight *K. aerogenes *suspension grown in LB medium usually yields 20 mL of an OD_600_ of around 100. **CAUTION:** To ensure that the prepared bacteria are a monoculture of *K. aerogenes*, perform all steps under a hood.
3. **Preparation of H40 electroporation buffer**
  1. Prepare 100 mL of buffer solution, dissolve 0.952 g of HEPES in ddH_2_O water, and add 100 µL of MgCl_2_ from a 1 M stock solution. Adjust to pH 7 using KOH for titration. Sterilize the buffer using a 0.22 µm filter or autoclave. Use acid-free HEPES and not the sodium salt.
4. Perform plasmid preparation following the manufacturer's protocol and using the kits summarized in the **Table of Materials**. Use the plasmids summarized in **Table 1**. **NOTE: **The quality of DNA used for transfection is crucial. The selection of *D. discoideum* transfectants growing on bacteria has specific requirements for the promoters driving the selection and expression cassette (see discussion).

**Table:** 

**plasmid name**	**resistance/selection in bacteria**	**resistance/selection in Dictyostelium**	**tag**
**extrachromosomal expression plasmids**
pDM1203	Ampicilin	G418	no
pDM1207	Ampicilin	G418	N-terminal GFP
pDM1208	Ampicilin	G418	N-terminal mCherry
pPI159	Ampicilin	G418	N-terminal mNeon
pPI437	Ampicilin	G418	N-terminal mScarlet
pPI54	Ampicilin	G418	N-terminal mTurquoise2
pDM1209	Ampicilin	G418	C-terminal GFP
pDM1210	Ampicilin	G418	C-terminal mCherry
pPI143	Ampicilin	G418	C-terminal mNeon
pPI459	Ampicilin	G418	C-terminal mScarlet
pPI142	Ampicilin	G418	C-terminal mTurquoise2
**shuttle plasmids**
pDM344	Ampicilin	no	no
pDM1019	Ampicilin	no	N-terminal GFP
pDM1018	Ampicilin	no	N-terminal mCherry
pPI152	Ampicilin	no	N-terminal mNeon
pPI418	Ampicilin	no	N-terminal mScarlet
pPI150	Ampicilin	no	N-terminal mTurquoise2
pDM1021	Ampicilin	no	C-terminal GFP
pDM1020	Ampicilin	no	C-terminal mCherry
pPI153	Ampicilin	no	C-terminal mNeon
pPI457	Ampicilin	no	C-terminal mScarlet
pPI151	Ampicilin	no	C-terminal mTurquoise2
**inducible extrachromosomal expression plasmids**
pDM1038	Ampicilin	Hygromycin	no
pDM1047	Ampicilin	Hygromycin	N-terminal GFP
pDM1046	Ampicilin	Hygromycin	N-terminal mCherry
pPI450	Ampicilin	Hygromycin	N-terminal mNeon
pPI452	Ampicilin	Hygromycin	N-terminal mScarlet
pPI449	Ampicilin	Hygromycin	N-terminal mTurquoise2
pDM1049	Ampicilin	Hygromycin	C-terminal GFP
pDM1048	Ampicilin	Hygromycin	C-terminal mCherry
pPI470	Ampicilin	Hygromycin	C-terminal mNeon
pPI460	Ampicilin	Hygromycin	C-terminal mScarlet
pPI469	Ampicilin	Hygromycin	C-terminal mTurquoise2
***act5* safe haven targeting plasmids**
pDM1501	Ampicilin	Hygromycin	no
pDM1513	Ampicilin	Hygromycin	N-terminal GFP
pDM1514	Ampicilin	Hygromycin	N-terminal mCherry
pPI231	Ampicilin	Hygromycin	N-terminal mNeon
pPI419	Ampicilin	Hygromycin	N-terminal mScarlet
pPI228	Ampicilin	Hygromycin	N-terminal mTurquoise2
pDM1515	Ampicilin	Hygromycin	C-terminal GFP
pDM1516	Ampicilin	Hygromycin	C-terminal mCherry
pPI230	Ampicilin	Hygromycin	C-terminal mNeon
pPI458	Ampicilin	Hygromycin	C-terminal mScarlet
pPI229	Ampicilin	Hygromycin	C-terminal mTurquoise2
**REMI expression plasmids**
pDM1220	Ampicilin	Hygromycin	no
pDM1351	Ampicilin	Hygromycin	N-terminal GFP
pDM1259	Ampicilin	Hygromycin	N-terminal mCherry
pPI465	Ampicilin	Hygromycin	N-terminal mNeon
pPI468	Ampicilin	Hygromycin	N-terminal mScarlet
pPI466	Ampicilin	Hygromycin	N-terminal mTurquoise2
pDM1352	Ampicilin	Hygromycin	C-terminal GFP
pDM1305	Ampicilin	Hygromycin	C-terminal mCherry
pPI471	Ampicilin	Hygromycin	C-terminal mNeon
pPI467	Ampicilin	Hygromycin	C-terminal mScarlet
pPI472	Ampicilin	Hygromycin	C-terminal mTurquoise2
**targeted in frame plasmids**
pDM1355	Ampicilin	Hygromycin	C-terminal GFP
pPI461	Ampicilin	Hygromycin	C-terminal mCherry
pPI462	Ampicilin	Hygromycin	C-terminal mNeon
pPI464	Ampicilin	Hygromycin	C-terminal mScarlet
pPI463	Ampicilin	Hygromycin	C-terminal mTurquoise2
**knock-out plasmids**
pDM1079	Ampicilin	Blasticidin	no
pDM1080	Ampicilin	Nourseothricin	no
pDM1081	Ampicilin	Hygromycin	no
pDM1082	Ampicilin	G418	no
**CRE expression plasmids**
pDM1483	Ampicilin	Nourseothricin	no
pDM1489	Ampicilin	Hygromycin	no
pDM1488	Ampicilin	G418	no

**Table 1: Plasmid list for non-axenic transfections.**

1. **Setting up *Dictyostelium* cells for transfection**
  1. Grow *K. aerogenes* to confluence in SM medium (nutrient-rich medium) overnight at RT. Cultures can be stored for up to 2 weeks at 4 °C.
  2. Add about 400 µL of this bacterial suspension onto a SM agar plate (peptone 10 g/L; yeast extract 1 g/L; glucose 10 g/L; KH_2_PO_4_ 1.9 g/L; K_2_HPO_4_ x 3 H_2_O, 1.3 g/L; MgSO_4_ anhydrous 0.49 g/L; 1.7% agar) and spread evenly. Take a sterile loop and inoculate with *Dictyostelium* cells. Spread the cells at one edge of the plate.
  3. Incubate the plate at 22 °C for 2 days to ensure sufficiently large growth zones for transfection. **NOTE: **For *Dictyostelium* strains that do not make large growth zones (*e.g., *Ax3, DH1, or JH10), or for inexperienced experimenters, use clearing plates instead. For this, follow the instructions in steps 1.5.4 to 1.5.6.
  4. Take a sterile loop and inoculate with *Dictyostelium* cells (approximately 2-4 x 10^5^ cells). Transfer the cells to 800 µL of a dense *K. aerogenes *suspension in SM. Mix cells by pipetting up and down.
  5. Transfer 400 µL, 200 µL, 100 µL, and 50 µL on fresh SM agar plates. Add to every plate 400 µL of additional SM *K. aerogenes *suspension, spread evenly, and dry.
  6. Incubate the plates at 22 °C for about 2 days until the plates become translucent. **NOTE: **Due to their faster growth rate, the bacteria initially produce a confluent lawn, and the amoebae subsequently "clear" the plate of the bacteria. The time required for this process can differ depending on the strain background and ability of mutant cells to grow on bacteria.

### 2. Transfection of *Dictyostelium* Cells Based on Bacterial Selection

**Figure:**
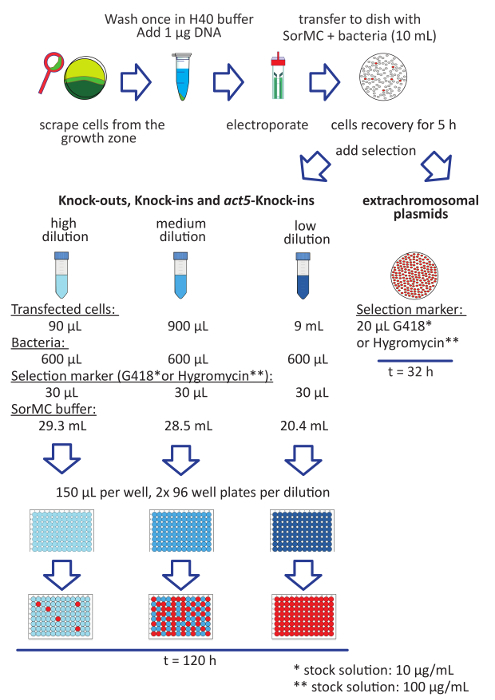

Figure 1**: Workflow for the transfection of bacteria-grown *Dictyostelium* cells.** The steps for transfection are listed as follows. Grow *D. discoideum cells* on a SM plate seeded with *K. aerogenes* bacteria (red). Harvest cells only from the feeding front (green), avoiding cells that are already developing (dark green). Wash the cells in H40. Resuspend cells to a final density of 2-4 x 10^7^ cells/mL. Mix the cell suspension with 1-2 µg of DNA. Transfer the mixture to an electroporation cuvette and pulse cells. Transfer the cells directly after electroporation to a dish with SorMC and bacteria. Allow the cells to recover for 5 h before adding the selectable marker. For extrachromosomal plasmids, add the selection directly to the dish. Transfectants are usually visible after ~2 days. For linearized constructs that aim for single integration into the genome, set up three dilutions as indicated and add the selection. Bacteria are taken from the OD_600_ = 100 stock solution. Mix the tubes well and transfer the cells into 96-well flat-bottom tissue culture plates. Use two plates per dilution. Pipette 150 µL of cell suspension into every well. It takes about 5 days until tight colonies are visible. The red wells show an example of the usual amount of successfully transformed cells obtained (upper panel modified from previous publication^22^). Please click here to view a larger version of this figure.

1. To prepare plates with *K. aerogenes* suspension, add 10 mL of SorMC buffer containing *K. aerogenes* bacteria to a density of OD_600_ = 2 (add 200 µL of the prepared OD_600 _= 100 *K. aerogenes* stock solution for the desired bacteria concentration) into a 10 cm tissue culture treated petri dish. **NOTE: **This plate is later needed to cultivate the transfected *D. discoideum* cells. Alternatively, a 6-well tissue-culture plate can be used. In the case of transfection of the extrachromosomal plasmids, the 6-well tissue-culture plate is more resource-efficient. Use 2 mL of *K. aerogenes* SorMC (OD_600_ = 2) suspension per well.
2. **Preparation of *Dictyostelium* cells**
  1. Using a 10 µL disposable inoculation loop, scrape cells from the growth zones (approximately 3 cm) of the culture plate (edge of the cleared area) or clearing plate. Transfer cells into a 1.5 mL tube containing 1 mL of ice-cold H40 buffer. NOTE: The timing of harvesting cells from clearing plates is crucial. Harvesting too early yields too little an amount of cells, while harvesting to late increases the risk of yielding partially developed cells.
  2. Wash the cells by spinning down for 2 min at 1,000 x *g* or flash-spinning for 2 s at 10,000 x *g*. Discard the supernatant and resuspend cells in H40 buffer to a final density of 2-4 x 10^7^ cells/mL. Keep the cells cold during the whole transfection procedure. Use an ice-water slurry to ensure direct contact of the tubes and ice.
3. **Electroporation**
  1. Add 100 µL of cells to a tube with 1-2 µg of DNA. Mix carefully by pipetting up and down.
  2. Transfer the cell/DNA mixture into a pre-chilled electroporation cuvette (2 mm gap).
  3. Pulse the cells using the following square-wave settings: 350 V, 8 ms, 2 pulses, and 1 s pulse interval. **NOTE:** Do not add more than 2 µg of DNA. Higher amounts are toxic to cells and decrease transfection efficiency. The total added DNA volume should not exceed 5 µL.
  4. Transfer cells immediately to the earlier prepared 10 cm Petri dish with *K. aerogenes *SorMC and allow the cells to recover for 5 h. **NOTE:** Check the cells in the Petri dish under an inverted microscope. Cells will appear round directly after electroporation but will return to their amoeboid shape after about 30 min, when they have had enough time to attach properly to the surface.
4. **Selection of transfectants: depending on whether transfection is aimed to generate a knock-out, knock-in, or *act5* knock-in, or to express a fluorescent reporter protein from an extrachromosomal plasmid, carry out the selection process through one of the following described methods.NOTE: **Unlike axenically grown cells, bacterially grown cells are highly resistant to blasticidin. Selection, therefore, is always carried out using G418 or hygromycin (see discussion).
  1. Knock-outs, knock-ins, and *act5 knock-ins*
    1. Detach the cells carefully from the Petri dish by repeatedly forcing the liquid from a pipette onto the surface.
    2. Set up three dilutions in the SorMC *K. aerogenes* suspension (OD_600_ = 2) and add the selective agent according to the resistance used (see Figure 1).
    3. Low dilution: mix 9 mL of cell suspension with 20.4 mL of SorMC and 600 µL of *K. aerogenes* stock solution.
    4. Medium dilution: mix 900 µL of cell suspension with 28.5 mL of SorMC and 600 µL of *K. aerogenes* stock solution.
    5. High dilution: mix 90 µL of cell suspension with 29.3 mL of SorMC and 600 µL of *K. aerogenes* stock solution.
    6. Add 30 µL of selectable marker (100x stock solution).
    7. Distribute the prepared dilutions into 96-well flat-bottom tissue culture plates by pipetting 150 µL of cell suspension into each well. **NOTE:** This procedure aims to screen single clones rather than populations. The selection takes about 5-7 days, depending on the construct used.
  2. Extrachromosomal plasmids
    1. Add the selectable marker directly to the 10 mL dish (see Figure 1). NOTE: There is no need to set up dilutions, since there is no desire for clonal populations. The selection process for extrachromosomal plasmids is faster due to high copy numbers present in *D. discoideum *cells. Transfectants can be expected after 32 h to 2 days. CAUTION: The antibiotics used as selectable marker are toxic. Wear gloves.
5. **Screen the obtained clones for positive tranfectants. For knock-out or knock-in attempts, follow the instructions in step 2.5.1. Check transfection success for extrachromosomal plasmids using the instructions in step 2.5.2.**
  1. For knock-outs, knock-ins, and *act5 *knock-ins, perform the initial screen via PCR to confirm integration of the construct into the correct genomic locus. **NOTE:** To maximize the likelihood of clonal populations, use the highest dilution possible that yields transfectants after selection. Aim for plates that are a maximum one-third of wells occupied.
    1. To expand clonal populations, transfer clones that have grown up after selection from the 96-well tissue-culture plate into a 12-well tissue-culture plate to grow enough cells for the isolation of genomic DNA. Supply each well with 1 mL of SorMC *K. aerogenes* (OD_600_ = 2) and fresh selectable marker. **NOTE:** 1 day is usually sufficient to obtain a confluent well suitable for DNA isolation.
    2. To perform mini genomic DNA isolation, harvest the cells of a confluent well and isolate genomic DNA using a mini DNA extraction kit following the manufacturer's instructions.
    3. Use the isolated genomic DNA together with suitable primers and *Taq*-polymerase (see **Table of Materials**) to PCR screen for positive integrands. **NOTE: **After confirmation of positive integration into the correct genomic locus, a Southern blot analysis should be performed. This ensures greater confidence that additional insertion events in unspecific genomic regions have not occurred.
  2. For fluorescent reporter proteins, visually identify positive fluorescent cells and check with a fluorescence microscope. Alternatively, perform a western blot with suitable antibodies for biochemical identification.

**Table:** 

**PCR program**
step	temperature	time
Initial Denaturation	94 °C	30 s
30 Cycles	94 °C	15-30 s
	42 °C	15-60 s
	68 °C	1 min/kb
Final Extension	68 °C	5 min
Hold	4-10 °C	
**Reaction composition**
component	25 μL reaction	final concentration
10 µM Forward Primer	0.5 µL	0.2 µM
10 µM Reverse Primer	0.5 µL	0.2 µM
Template DNA	variable (ca. 5 µL)	< 1,000 ng
2x Master Mix with Standard Buffer including polymerase (see table of materials)	12.5 µL	1x
Nuclease-free water	to 25 µL	< 1,000 ng

**Table 2: PCR program and sample composition for the amplification of** ***D. discoideum*** **genomic DNA.**

## Representative Results

Extrachromosomal plasmids are used for reporter studies, which aim to identify the localization of certain proteins inside a cell or changes in cellular structure of mutant cells. For many approaches, such as monitoring of the cell cycle, it is crucial to express two reporters at the same time. This is now possible using our dual reporter extrachromosomal plasmid system (**Table 1**). On day 1, cells were transfected before adding the selectable marker G418 after 5 h (Figure 1). In the example, NC4, DdB, Ax2, and the independently derived wild isolates V12M2 and WS2162 (**Supplementary Table 1**) were transfected with the plasmid pPI289, which encodes for GFP-TubulinA, a marker for microtubules and mCherry-PCNA, a protein that is used for monitoring the cell cycle (Figure 2**A**). After 32 h, the cells were observed under the microscope. The majority of cells expressed both fluorescent-labeled fusion proteins, consistent with previous reports that expression of two reporters from the same plasmid shows similar expression levels, which is nearly impossible when using two different plasmids^18^,^19^. A representative cell for each cell line (NC4, DdB, Ax2, V12M2, and WS2162) expressing the desired dual reporter is shown in Figure 2**B**. The transfection efficiencies are summarized in **Figure2C**. NC4-derived cell lines show the best transfection efficiencies. However, for cell lines V12M2 and WS2162, a considerably high number of transfectants was obtained.

**Figure:**
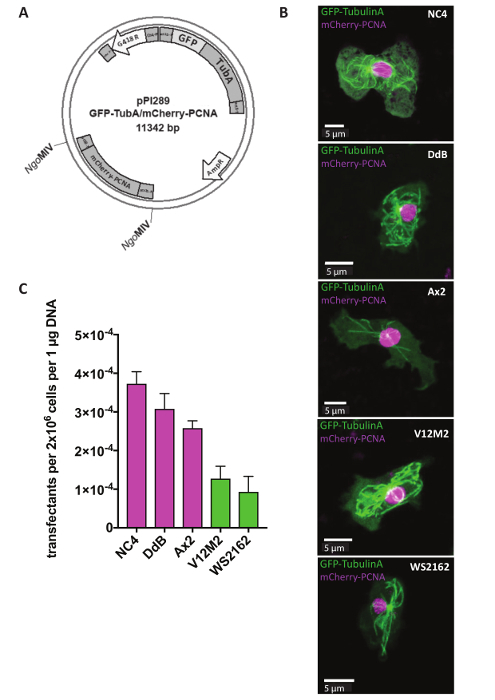

Figure 2**: Expression of an extrachromosomal plasmid.** (**A**) Extrachromosomal plasmids are directly transfected in circular form. As an example, the dual reporter pPI289 is shown. The *Ngo*MIV sites indicate insertion of the second reporter into the extrachromosomal expression plasmid.** (B**) Z-projection of a representative cell expressing GFP-TubulinA (cytoplasmic) and mCherry-PCNA (largely nuclear) for five different wild-type cell lines used (NC4, DdB, Ax2, V12M2, WS2162). (**C**) The transfection efficiencies for the five cell lines pictured in (B) were calculated. Shown is the mean of two experiments. Error bars indicate ± SD. Please click here to view a larger version of this figure.

Integration of a targeting vector into a specified genomic locus is more challenging and requires more careful analysis of the generated cell line. In Figure 3, an *act5*-mCherry KI in NC4 is attempted. First, the plasmid must be linearized to increase the frequency of recombination events following transfection. For this, the plasmid pDM1514 is cut with *Ngo*MIV. Two bands are obtained after running the digest on an agarose gel. The 4127 bp band contains the desired construct (Figure 3). For transfection, the digested DNA must be extracted from the gel and purified using a gel extraction kit following the manufacturer's instructions.

**Figure:**
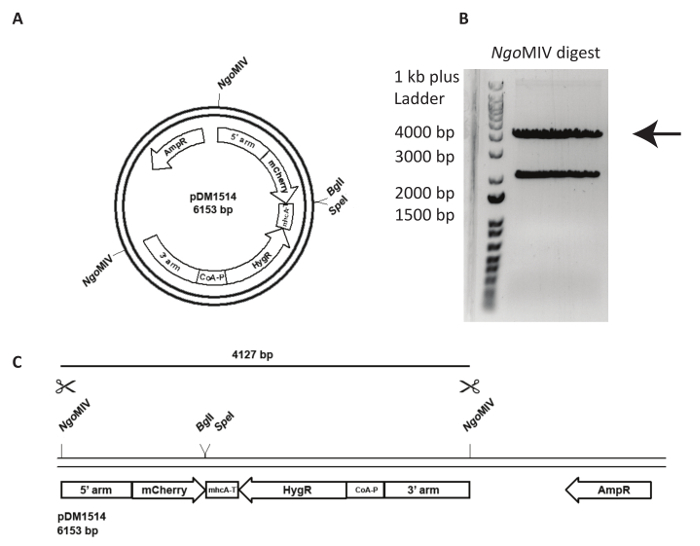

Figure 3**: Preparation of *act5* knock-in and DNA for transfection.** An example of the use of an *act*5 knock-in the plasmid pDM1514 is shown. The steps for preparation are listed as follows. (**A**) Before electroporation, linearize the plasmid using the indicated *Ngo*MIV sites. (**B, C**) Run the cut plasmid on an agarose gel until the two expected bands are properly separated. Cut out the 4127 bp band containing the recombination arms, mCherry, and the resistance-cassette, and gel extract the DNA. The DNA is now ready for transfection. Please click here to view a larger version of this figure.

The purified DNA was used for transfection of NC4 cells. After 5-6 days of selection, clones were obtained. Representative transfection efficiencies and the amount of positive identified clones for several *act5* knock-in attempts are summarized in **Table 3**. Two clones of the NC4 transfection were randomly chosen and analyzed by PCR (Figure 4**A**), and both showed the predicted band patterns expected of a knock-in and were further validated with Southern blot analysis to ensure a single integration event of the construct into the genome^20^. The blot shows a clear single integration in the desired *act5* locus (Figure 4**B**). The generated *NC4::act5-mCherry* cell line can now be used in experiments.

**Figure:**
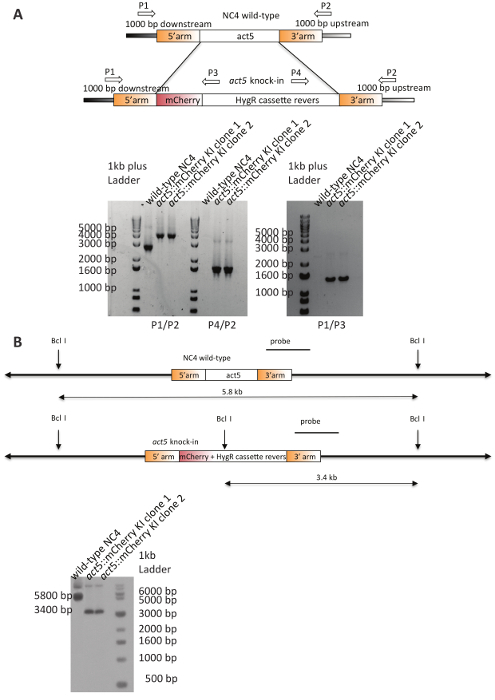

Figure 4**: Validation of *act5*-mCherry KIs in NC4.**(**A**) Scheme and control PCRs for the validation of positive integrations into *act5* locus. The indicated primers were used to analyze two independent clones and the parent. Both clones show the expected bands for the resistance-cassette and downstream (P1) or upstream primer (P2), which are not present in the parental strain. The primer combination (P1/P2) confirms the correct integration of mCherry and resistance cassette into the *act5* locus. The wild-type NC4 shows the expected 2800 bp band, while both KI clones lack this band and instead display a PCR product about 1400 base pairs larger. (**B**) Scheme for the used restriction digest and Southern blot. Both knock-in clones show the smaller 3400 bp band, which is the result of integration of the construct into the *act*5 locus, specifically the additional *Bcl*I site in the hygromycin resistance cassette. The wild-type control shows the expected 5.8 kb resulting from the two downstream located *Bcl*I sites. The blot was cropped and spliced for clarity. Please click here to view a larger version of this figure.

**Table:** 

**targeted construct into the act5 locus**	**plasmid name**	**number of occupied wells**	**number of checked clones**	**Positive clones**	**Correct clones (%)**	**Dictyostelium strain used**	**transfection efficiency (transfectants/ 2x10^6/1 µg DNA)**
LifeAct-mCherry	pPI226	7	7	1	14.2	AX2	3.5x10^-6
LifeAct-GFP	pPI227	12	12	5	41.6	AX2	6x10^-6
GFP	pDM1513	3	3	2	66.6	AX2	1.5x10^-6
mCherry	pDM1514	3	3	1	33.3	AX2	1.5x10^-6
GFP	pDM1513	66	9	5	55.5	AX2	3.3x10^-5
mCherry	pDM1514	221	12	10	83.3	AX2	1.1x10^-4
H2B-mCherry	pPI420	3	3	1	33.3	AX2	1.5x10^-6
H2B-mCherry	pPI420	7	7	6	85.7	AX2	3.5x10^-6
mCherry	pDM1514	10	10	7	70	DdB	5x10^-6
mCherry	pDM1514	240	12	11	91.6	DdB	1.2x10^-4
mCherry	pDM1514	320	12	12	100	NC4	1.6x10^-4

**Table 3: Transfection efficiencies and the amount of obtained positive transfectants for the generation of** ***act5*** **KIs in different strain backgrounds** **22** **.**

The *act*5 locus offers relatively homogenous expression of the integrated reporter^21^. The generated *NC4::act5-mCherry* cells allow mix experiments to be performed with other *act5* knock-ins using a different fluorescent protein such as GFP. To emphasize the great advantage of this system, mixing experiments with *Ax2::act5-GFP* are shown. Due to the inability to transfect non-axenic wild-type cells, this type of approach could not be performed before. Mix experiments are an important tool to analyze cell behavior, because they allow a direct comparison between different cell lines experiencing identical experimental conditions. NC4 cells grow faster on bacterial lawns than Ax2 cells (Figure 5**A**). This may be due to a higher capacity to phagocytose bacteria or improved ability to move and show chemotaxis towards a food source. Using an under-agar folate chemotaxis assay, direct comparison of a population of *NC4::act5-mCherry* and *Ax2::act5-GFP* was performed, showing that *NC4::act5-mCherry* are much more efficient in folate sensing. After 4 h, more *NC4::act5-mCherry* cells were able to crawl under the agarose than *Ax2::act5-GFP* cells (Figure 5**B**). Analyzing standard metrics of chemotaxis for the cells migrating under the agarose revealed that *NC4::act5-mCherry* cells were faster and showed stronger chemotactic response than *Ax2::act5-GFP* cells (Figure 5**C-E**).

**Figure:**
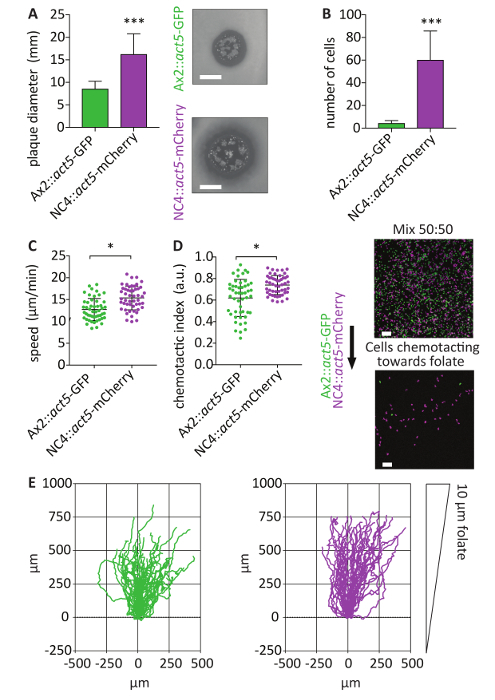

Figure 5**: Using *act*5 KIs for image-based chemotaxis mix experiments.** (**A**) *NC4::act5-mCherry* and *Ax2::act5-GFP* expressing the indicated fluorescent protein from the *act*5 locus were analyzed for the ability to grow on a bacterial lawn. After 4 days, the plaque diameter arisen from solitary plated *Dictyostelium* cells were measured. Non-axenic *act5*::NC4 cells make significantly bigger plaques than axenic *Ax2::act5-GFP* cells (mean ± SD, ***p < 0.0001, n = 3, scale bar 5 mm). **(B**) For use of the under-agarose folate chemotaxis assay^22^ to directly compare the chemotactic abilities of the *NC4::act5-mCherry* and *Ax2::act5-GFP* used in (A), bacteria- grown amoebae of both strains were mixed in a 50:50 ratio. Cells were allowed to crawl under the agarose. After 4 h, cells that were migrating up the folate gradient were imaged using a confocal microscope. The number of *NC4::act5-mCherry* and *Ax2::act5-GFP* cells was then determined. *NC4::act5-mCherry* cells were more efficient in sensing folate. About 10-fold more *NC4::act5-mCherry* cells were found compared to *Ax2::act5-GFP* cells (mean ± SD, ***p < 0.0001, n = 6, scale bar 100 µm). (**C, D**) The cells were filmed for 60 min, and their speed and chemotactic index were calculated. After pre-selection of the most chemotactically responsive cells (only those that migrated under the agarose), *Ax2::act5-GFP* cells showed lower values for both cell speed and chemotaxis. Fifty cells per cell line were analyzed. (median ± SD, *p < 0.01, n = 3). (**E**) The tracks of *NC4::act5-mCherry* and *Ax2::act5-GFPact*5 knock-ins are plotted over 60 min, showing the more directed movement towards the chemoattractent source of the *NC4::act5-mCherry* cells (median ± SD, ***p < 0.0001, n = 6). Please click here to view a larger version of this figure.

The *act5* knock-in cell lines can also be used for flow cytometry. As with growth on bacteria, there are major differences in development between axenic strains and non-axenic wild-types. After development, the fruiting bodies of NC4 cells are approximately twice as big as those derived from Ax2 cells. If cells are mixed, an intermediate sized fruiting body is obtained. To analyze the contribution of the both cell lines more quantitatively, flow cytometry was used. These analyses showed clearly that the intermediate-sized fruiting bodies are due to different levels of contribution from both cell lines. While *NC4::act5-mCherry* cells made up about 75% of the measured spores, *Ax2::act5-GFP* contributed only 25%, revealing a potential fitness advantage for non-axenic strains (Figure 6). Since the analysis does not monitor the stalk cell population, there are two possibilities to explain the imbalance in development between Ax2 and NC4. One possibility is that Ax2 cells contribute mainly to the stalk cell population, rather than entering the spore cell population. Alternatively, more NC4 cells may enter the developmental cycle, with Ax2 relatively delayed, and they are consequently unable to contribute to the assembly of a fruiting body. The possibility to transfect non-axenic wild-type cells further develops other approaches and greatly simplifies experimental procedures.

**Figure:**
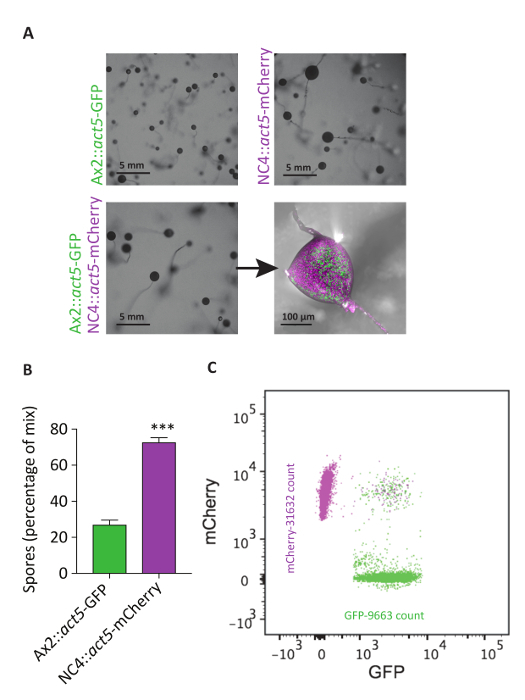

Figure 6: ***Act*****5 KIs allow analysis of mix experiments using flow cytometry. **(**A**) *NC4::act5-mCherry* and *Ax2::act5-GFP* were developed separately or in a 50:50 mixture on non-nutrient agar plates. *NC4::act5-mCherry* cells form larger fruiting bodies than *Ax2::act5-GFP*. The mix instead shows an intermediate size (scale bar 5 mm). Confocal fluorescence light microscopy suggests a higher amount of *NC4::act5-mCherry* spores in the spore heads derived from mixes. (**B**) To quantify this observation, the amounts of spores in harvested spore heads from the mixing experiment in (A) were analyzed by flow cytometry. About 75% of the spores originated from *NC4::act5-mCherry* cells, with only 25% from *Ax2::act5-GFP* cells. (**C**) Representative distribution of spores from both cell lines shown in a flow cytometry scatter. About 0.05% show positive mCherry and GFP signals, suggesting that parasexual fusion processes have occurred or that spores are sticking to each other. Please click here to view a larger version of this figure.

**Table:** 

**Cell line**	**Genetically background**	**Reference number**	**Published before**	**Type**
AX2 (Ka)		DBS0235521	Bloomfield et al., 2008	wild type
NC4 (S)			Bloomfield et al., 2008	wild type
act5::mCherry Clone 5	NC4	HM1912	Paschke et al., 2018	act5 knock-in
act5::GFP Clone 2	AX2	HM1930	Paschke et al., 2018	act5 knock-in
V12M2			Bloomfield et al., 2008	wild type
WS2162			Bloomfield et al., 2008	wild type

**Supplementary Table 1: Different strains of** ***Dictyostelium*** **studied.**

## Discussion

The use of non-axenic, wild-type *Dictyostelium* cells has been very limited so far in molecular research. Available methods for genetic engineering of these strains have lacked reliability and efficiency^23^, preventing their general adoption. The generated materials and protocols presented here can be used for any *D. discoideum* strain independent of its ability to grow in liquid medium. It should be mentioned that this protocol is optimized for NC4-derived cell lines. Transfection efficiencies for freshly isolated strains from the wild differ from NC4, as we have observed before and are shown here for V12M2 and WS2162^8^,^9^. The electroporation conditions seem in particular to have a considerable influence on transfection efficiencies and may require further optimization for some strains. In general, a sufficient amount of transfectants have been observed in all strains tested so far, showing that these methods are workable. The number of positive transfectants obtained using NC4-derived strains is higher compared to other non-axenic wild-type strains, but in all cases, sufficient numbers of transfectants are obtained to allow for further experiments. This, plus the simplicity of our protocol, is the major improvement compared to earlier attempts^8^,^9^.

These new non-axenic protocols cover all standard genetic procedures and add further advantages, since transfections can be performed rapidly and efficiently in parallel. Positive clones can be obtained in days rather than weeks, since growth on bacteria halves the division time. The newly established plasmids also work under axenic conditions and can be routinely used under both growth conditions, which is another advancement of this methology^22^. As introduced in the protocol, the plasmid used for selection on bacteria have special requirements that are crucial for the success of the transfection. In particular, the promoters driving the expression and resistance cassettes are important for successful transfection. The often used *act6* (actin 6) promoter^24^ for driving the resistance or expression cassettes lacks efficiency when cells are grown on bacteria. In our plasmid system, the highly active *act15* (actin 15) promoter drives all expression cassettes, while the resistance cassettes are under the control of a *coA* (coactosin A) promoter, both of which are active under axenic conditions and in cells grown on bacteria. Requirements for the expression of reporter constructs as well as knock-in and knock-out constructs make it necessary to use our plasmid repertoire, but unfortunately limits the use of vectors already created that use the inefficient *act6* promoter.

Promoter efficiencies are especially critical for transfections that depend on a single integration event into the correct genomic locus. Due to our improvement of promoters driving the resistance gene expression, enough resistance protein is produced from single locus integrations. A major concern was the favoring of multiple integrations into the genome when using hygromycin or G418 as described. No favoring of multiple integrations has been observed so far, as reported previously for G418 selections using older promoter systems^25^. This means that both hygromycin and G418 are suitable selectable markers for the generation of clean knock-outs and knock-ins. Unfortunately, the selectable marker blasticidin does not work under non-axenic conditions. This is a major disadvantage of our method, since the blasticidin resistance cassette is routinely used to generate knock-out constructs in *D. discoideum*. Constructs that were already generated with blasticidin resistance cassettes will need to be rederived using one of the workable selectable markers. Another possibility to overcome this limitation is to combine the recently established axenic *D. discoideum* CRISPR technology with this transfection protocol^26^. The generation of suitable, single guide RNAs (sgRNAs) is simpler and faster than the reconstruction of complete knock-out constructs. For future directions, the possibility of generating multiple knock-outs using CRISPR/Cas9 combined with this transfection protocol is appealing and may pave the way for many researchers in the *Dictyostelium* community. However, the workability of the established transient expression system used for CRISPR/Cas9 in axenic grown cells should be carefully examined.

The *act5* knock-in system presented offers a reliable and safe integration system for the generation of stable cell lines in *D. discoideum,* with the advantage of similar sites established in other organisms^22^. It also depends on a single integration event in the genome and offers many possibilities for different research directions. The *act5* promoter is strongly active and guarantees almost homogenous expression^27^ independent of the cultivation conditions. Fluorescent reporter proteins can easily be integrated into this safe haven locus using the engineered targeting plasmids. This can be useful, for example, for cell-tracking purposes, as shown here in a mix experiment. The cells show minimal cell-to-cell expression variability, which can assist in automated cell tracking. Importantly, the insertion of a desired sequence into the *act*5 locus appears phenotypically neutral^28^,^29^. As the expression does not depend on a selectable marker to maintain protein expression, as seen in random integrants or extrachromosomal vector-bearing cell lines, the *act5* system can be useful for rescue experiments, as well. Since the cell lines are homogenous, all cells can be analyzed. This is not possible using an extrachromosomal plasmid for expression, in which there is considerable heterogeneity in expression.

In addition to these general improvements for all strains, the ability to use non-axenic wild-type cells for molecular research allows an assessment of the effects of accumulated mutations in current laboratory strains. Multiple genetic rearrangements has been found since the adoption of the Ax2 strain in 1970, as shown by previously published microarray analyses^26^. Synthetic phenotypes are observed because of the presence of these mutations. For example, a reported *phg*2 mutant shows decreased adhesion in one laboratory strain background and increased adhesion in another^3^. Such conflicting data can now be resolved by repeating the experiment in the common ancestor strain (in this case, DdB). The strength of this approach has been recently shown for the small GTPase RasS^30^,^31^.

The ability to perform genetic experiments in any *D. discoideum* strain expands the potential of new research directions that depend on the use of wild isolates, notably those involving social evolution, kin recognition, and the sexual cycle^22^.

## Disclosures

The authors have no conflicts of interest to disclose.
